# Pim kinases phosphorylate multiple sites on Bad and promote 14-3-3 binding and dissociation from Bcl-X_L_

**DOI:** 10.1186/1471-2121-7-1

**Published:** 2006-01-10

**Authors:** Andrew Macdonald, David G Campbell, Rachel Toth, Hilary McLauchlan, C James Hastie, J Simon C Arthur

**Affiliations:** 1 MRC Protein Phosphorylation Unit, School of Life Sciences, University of Dundee, Dundee, DD1 5EH, UK; 2 Division of Signal Transduction Therapy, School of Life Sciences, University of Dundee, Dundee, DD1 5EH, UK

## Abstract

**Background:**

Pim-1, 2 and 3 are a group of enzymes related to the calcium calmodulin family of protein kinases. Over-expression of Pim-1 and Pim-2 in mice promotes the development of lymphomas, and up-regulation of Pim expression has been observed in several human cancers.

**Results:**

Here we show that the pim kinases are constitutively active when expressed in HEK-293 cells and are able to phosphorylate the Bcl-2 family member Bad on three residues, Ser112, Ser136 and Ser155 *in vitro *and in cells. *In vitro *mapping showed that Pim-2 predominantly phosphorylated Ser112, while Pim-1 phosphorylated Ser112, but also Ser136 and Ser155 at a reduced rate compared to Ser112. Pim-3 was found to be the least specific for Ser112, and the most effective at phosphorylating Ser136 and Ser155. Pim-3 was also able to phosphorylate other sites in Bad *in vitro*, including Ser170, another potential *in vivo *site. Mutation of Ser136 to alanine prevented the phosphorylation of Ser112 and Ser155 by Pim kinases in HEK-293 cells, suggesting that this site must be phosphorylated first in order to make the other sites accessible. Pim phosphorylation of Bad was also found to promote the 14-3-3 binding of Bad and block its association with Bcl-X_L_.

**Conclusion:**

All three Pim kinase family members predominantly phosphorylate Bad on Ser112 and in addition are capable of phosphorylating Bad on multiple sites associated with the inhibition of the pro-apoptotic function of Bad in HEK-293 cells. This would be consistent with the proposed function of Pim kinases in promoting cell proliferation and preventing cell death.

## Background

Pim-1, 2 and 3 make up a group of closely related mammalian serine/threonine kinases that form part of the calmodulin dependent protein kinase family [1,2]. Pim-1 was first identified as an oncogenic gene in murine T-cell lymphomas, which became activated after pro-viral insertion in the 3'UTR of the Pim-1 gene [3]. Insertion in this region stabilised the mRNA for Pim-1 thus up-regulating the protein [4]. Over-expression of the Pim-1 gene in B and T cells in transgenic mice resulted in a high level of lymphoma development in these mice, confirming the oncogenic properties of this protein [5,6]. A second related gene, Pim-2, was also identified as a common site of viral insertion in murine lymphomas [7]. Like Pim-1 the over-expression of Pim-2 in transgenic mice predisposes the mice to the development of lymphoma [8]. The precise mechanism by which Pim-1 and 2 promote the formation of lymphomas is not fully understood, however it has been suggested that Pim-1 synergises with c-myc in this process [8-10]. Pim-1 also appears to be important in the development of human cancers, as the Pim-1 gene maps to an area showing karyotypic abnormalities in leukaemia, and the mRNA for Pim-1 has been shown to be up-regulated in prostate cancer and leukaemia [11-13]. A third isoform, Pim-3, which is 72% identical to Pim-1 also exists in mammalian cells although the oncogenic properties of this protein are less well studied. Pim-3 has recently been shown to be up-regulated in Ewing's tumour cell lines, is a transcriptional target for the oncogenic EWS transcription factors and is able to promote anchorage-independent cell growth in tissue culture models [14]. Recently, over-expression of Pim-3 has been demonstrated in a mouse model of hepatocellular carcinoma, although the functional role of Pim-3 was not determined [15].

Several substrates have been suggested for Pim-1 including mdm2 [16], p21 [17], cdc25 phosphatase [18] and c-myb [19], all proteins involved in control of the cell cycle. Pim-2 has been reported to maintain phosphorylation of the translational repressor 4EBP-1 and to phosphorylate and inactivate the pro-apoptotic protein Bad on Ser112 [20,21]. Pim-1 has also been reported to be able to phosphorylate Bad on Ser112 [22], however the ability of Pim-3 to phosphorylate Bad is unknown. Both Pim-1 and Pim-2 therefore appear to act as survival or proliferation signals *in vivo*. Consistent with this Pim-1 expression has been shown to promote cytokine independent growth of haematopoetic cells [23,24]. In contrast, less is known about the substrates of Pim-3, and its function *in vivo *is still largely unclear.

Bad is a pro-apoptotic member of the BH3 family of proteins. Active Bad induces apoptosis by binding to anti-apoptotic Bcl-2 family members such as Bcl-X_L_. This allows other pro-apoptotic proteins such as BAX and BAK to aggregate, inducing cytochrome C release from the mitochondria and caspase activation (reviewed in [25]). The activity of Bad is repressed by phosphorylation, and three residues (Ser112, Ser136 and Ser155) have been identified as the predominant *in vivo *phosphorylation sites. In addition to Pim-2 several kinases have been identified as potential kinases for Bad, including PKB [26-28], RSK [29,30], PAK [31], p70S6K [32] and PKA [33-35]. It seems likely that multiple kinases may phosphorylate Bad on these sites *in vivo*, depending on the cell type and situation. Consistent with this, several of the proposed kinases preferentially phosphorylate one or two of the three sites in Bad. For instance, *in vitro *RSK phosphorylates Ser112 and to a lesser extent Ser155, while PKB phosphorylates Ser136 and PKA preferentially phosphorylates Ser155 [33]. In addition to these phosphorylation sites, an unidentified kinase has been shown to inhibit apoptosis by phosphorylating Bad on Ser170 [36]. Conversely, phosphorylation by Cdc2 of Ser128 in Bad was found to promote the apoptotic activity of Bad [37].

In this paper we examine the specificity of Pim-1, 2 and 3 for the Ser112, 136 and 155 phosphorylation sites in Bad, both *in vitro *and in HEK-293 cells.

## Results

### Pim kinases phosphorylate Bad on three sites *in vitro*

**Figure 1:**
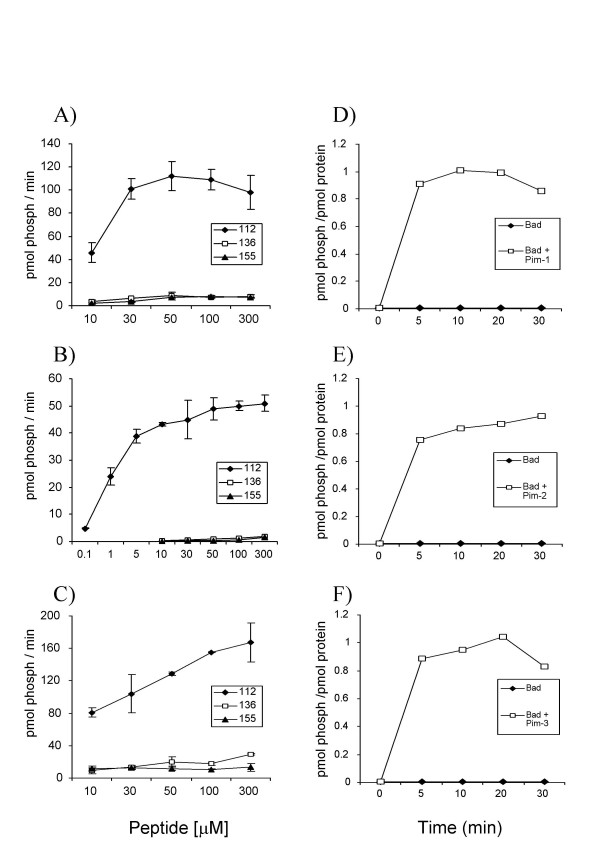
**Phosphorylation of peptides derived from Bad and recombinant Bad protein by Pim kinases**. A-C) Recombinant Pim-1 (A), Pim-2 (B) or Pim-3 (C) were incubated in *in vitro *kinase assays with the indicated concentrations of synthetic peptides from around the three potential phosphorylation sites in Bad as described in the methods. The peptides used were RSRMSSYPDRG (corresponding to Ser112), RGSRSAPPNL (corresponding to Ser136) and RELRRMSDEFEGS (corresponding to Ser155). After 10 min at 30°C, the reactions were terminated and the amount of phosphate incorporated into the peptide determined. D-F) Recombinant Pim-1 (D), Pim-2 (E) or Pim-3 (F) were used to phosphorylate 2 μg of Bad as described in the methods. At the times indicated reactions were stopped by the addition of SDS sample buffer and the amount of phosphate incorporated into Bad was determined by SDS gel electrophoresis and Cerenkov counting of the Bad band.

**Figure 2:**
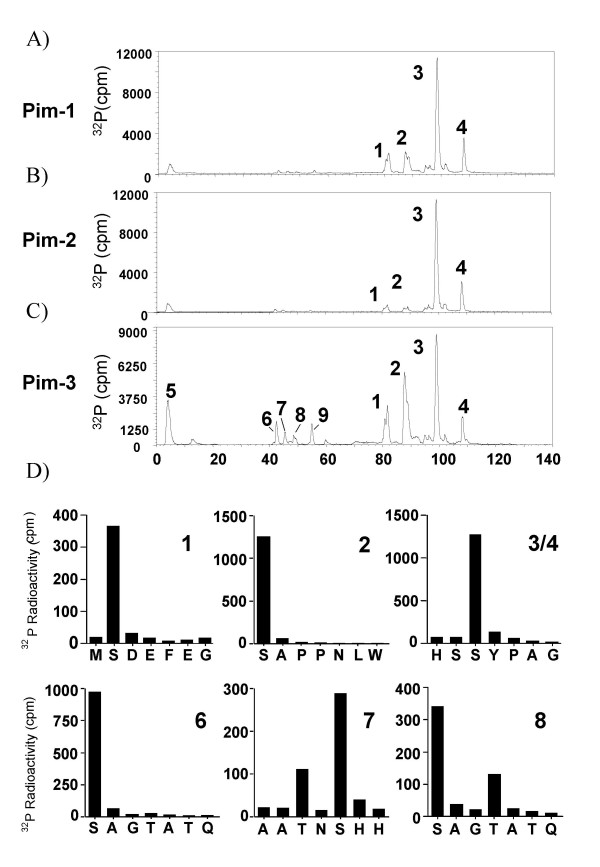
**Phospho site mapping of Pim phosphorylation sites in Bad**. Bad was phosphorylated by 10 mU of Pim-1 (A), Pim-2 (B) or Pim-3 (C) at 30°C for 10 min. Bad was then digested with trypsin as described in the methods and the resulting peptides resolved using an acetonitrile gradient on reverse phase HPLC. Phospho peptides were analysed by ms/ms and solid phase Edman sequencing to determine the phosphorylated residue. (D) Solid phase sequencing results for the peaks obtained with Bad phosphorylated by Pim-3. For each peptide, release of ^32^P was obtained within the first 7 cycles (see section D). Further cycles to the end of the peptides did not result in the release of more peaks of ^32^P (data not shown). Similar results were obtained for peaks 1 to 4 using Pim-1 and Pim-2 phosphorylated Bad (data not shown).

Bad has been described previously to be phosphorylated on three sites, Ser112, Ser136 and Ser155 *in vivo*. Peptides from around these sites were used as substrates for purified Pim-1, 2 or 3 in *in vitro *kinase assays. All three Pim kinases were found to phosphorylate the Ser112 peptide efficiently (Fig 1A–C). In contrast the Ser136 and Ser155 peptides were much less effective substrates for Pim-1 and Pim-3 (Fig 1A and 1C) and very poor substrates for Pim-2 (Fig 1B). As phosphorylation of synthetic peptides does not always give a true reflection of a kinases ability to phosphorylate the corresponding site in a protein, the ability of Pim kinases to phosphorylate recombinant Bad protein was also examined. As expected Bad was phosphorylated by all three Pim kinases, with maximum phosphorylation occurring in the first 5 to 10 min (Fig 1D–F). This activity was due to the Pim kinase, as incubation of the Bad protein in the absence of Pim (Fig D-F), or with kinase dead Pim (data not shown), did not result in any incorporation of ^32^P into Bad. To determine the sites phosphorylated by Pim-1, 2 or 3, recombinant Bad was phosphorylated with ^32^P labelled ATP and digested with trypsin. Peptides were then resolved by reverse phase HPLC and ms/ms used to identify the phospho peptides eluted (data not shown). The position of the phosphate in each peptide was also confirmed by solid phase sequencing reactions and measuring the cycle at which radioactivity was released. Pim-1 phosphorylated 4 major peptides in Bad, designated P1, P2 P3 and P4 (Fig 2A). Peak P1 was found to correspond to phospho Ser155, P2 to Ser136 while both P3 and P4 corresponded to phospho Ser112 (Table 1). The different elution times of the Ser112 peptides is probably explained by different oxidation states of the methionine residue in the peptide. Analysis of the cpm in each peak showed that Ser112 was the site most highly phosphorylated by Pim-1, with approximately 7 times the level of phosphorylation than occurred at either Ser136 or Ser155. This was consistent with what was found for the rate of phosphorylation of the synthetic peptides for each site (Fig 1A). Pim-2 was found to phosphorylate two major peaks that eluted at an identical position to the P3 and P4 peaks in the Pim-1 phosphorylated Bad (Fig 2B). Ms/ms analysis confirmed that these peptides also corresponded to Bad phosphorylated on Ser112. Small peaks were also seen corresponding to P1 and P2 in the Pim-2 phosphorylated Bad HPLC trace, and these were confirmed to correspond to phospho Ser155 and Ser136. The amount of phosphate incorporated at these sites was 15 times less than that at Ser112 (Table 1).

**Table 1:** Phosphorylation site analysis of Bad. Phosphorylated peptides were eluted from the HPLC (Fig 2) were identified by ms/ms mass spectrometry. The amino acid numbers of the peptide in murine Bad are indicated and the phosphorylation site underlined. Results for the release of ^32^P during Edman degradation cycle sequencing and phospho amino acid analysis of these fractions are also indicated. The percentage of ^32^P obtained in each HPLC peak, relative to the total ^32^P eluted in all the peaks is also listed (last column)

Kinase	Peak	Residue Numbers	Peptide Sequence	Phospho-amino acid analysis	Solid Phase cycle no.	Phosphorylation site	% 32P
Pim-1	1	154 – 163	MSDEFEGSFK	Phospho Ser	2	Ser 155	11
	2	136 – 146	SAPPNLWAAQR	Phospho Ser	1	Ser 136	15
	3	110 – 131	HSSYPAGTEEDEGMEEELSPFR	Phospho Ser	3	Ser 112	60
	4	110 – 131	HSSYPAGTEEDEGMEEELSPFR	Phospho Ser	3	Ser 112	14

Pim-2	3	110 – 131	HSSYPAGTEEDEGMEEELSPFR	Phospho Ser	3	Ser 112	82
	4	110 – 131	HSSYPAGTEEDEGMEEELSPFR	Phospho Ser	3	Ser 112	18

Pim-3	1	154 – 163	MSDEFEGSFK	Phospho Ser	2	Ser 155	14
	2	136 – 146	SAPPNLWAAQR	Phospho Ser	1	Ser 136	28
	3	110 – 131	HSSYPAGTEEDEGMEEELSPFR	Phospho Ser	3	Ser 112	27
	4	110 – 131	HSSYPAGTEEDEGMEEELSPFR	Phospho Ser	3	Ser 112	6
	5	unidentified	unidentified	unidentified	N/A	unidentified	13
	6	170 – 178	SAGTATQMR	Phospho Ser	1	Ser 170	4
	7	92 – 107	AATNSHHGGAGAMETR	Phospho Thr/Ser	3, 5	Thr 94 Ser 96	2
	8	170 – 178	SAGTATQMR	Phospho Ser/Thr	1, 4	Ser 170 Thr 173	2
	9	unidentified	unidentified	unidentified	N/A	unidentified	3

Pim-3 phosphorylated Bad also gave peaks corresponding to P1, P2, P3 and P4, and again these were confirmed as Ser112, Ser136 and Ser155 by ms/ms and solid phase sequencing (Fig 2C and 2D). Of the three kinases Pim-3 gave the highest phosphorylation of Ser136 and Ser155 compared to Ser112 in Bad (Table 1). Five further smaller phospho peptide peaks (P5-9) were also observed in the HPLC when Pim-3 was used to phosphorylate Bad. These sites were not however significantly phosphorylated by Pim-1 or Pim-2. P5 was observed in the flow through of the column and peptides in this fraction were not identified by mass spectrometry. Prediction from the Bad sequence shows that tryptic digest would give 2 small peptides that contain serine or threonine that could have been phosphorylated. Phosphopeptides from P9 were also not successfully identified by mass spectrometry. P7 was identified as the peptide AATNSHHGGAGAMETR (residues 92 to 107 of Bad) with 1 phospho group. Ms/ms analysis suggested that this was a mixture of phosphorylation on either Thr94 or Ser96. Solid phase sequencing confirmed this result, and also suggested that Ser96 was the predominant site (Fig 2D). P6 and P8 were identified by mass spectrometry as SAGTATQMR (residues 170 to 178 in Bad) phosphorylated at Ser170. The phosphorylation of Ser170 was confirmed by solid phase sequencing for both P6 and P8. Surprisingly, solid phase sequencing also showed that in P8 the 4^th ^residue in the peptide was also phosphorylated, suggesting that Thr 173 may also be phosphorylated by Pim-3.

### Pim kinases are constitutively active in HEK-293 cells and phosphorylate Bad on Ser112, 136 and 155

**Figure 3:**
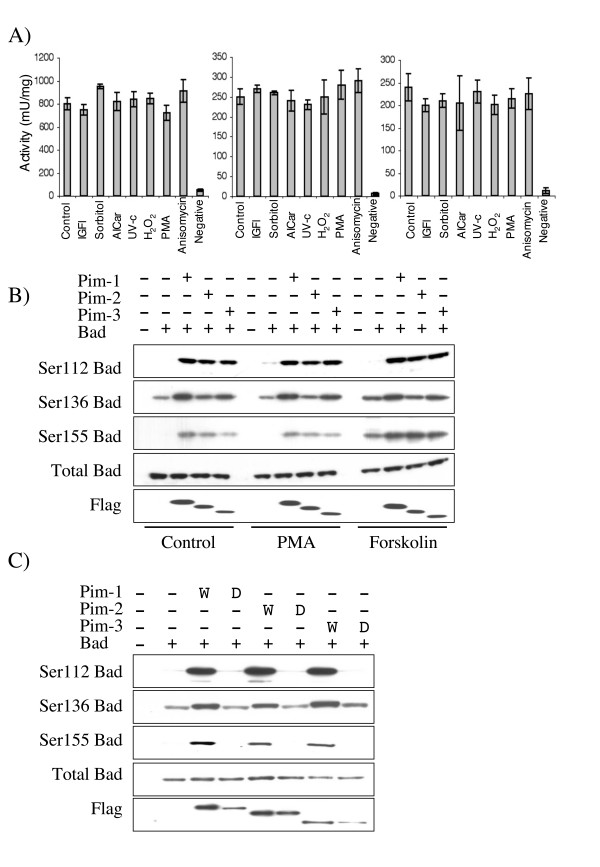
**Phosphorylation of Bad by Pim kinases in HEK-293 cells**. A) FLAG-Pim-1, Pim-2 or Pim-3 were expressed in HEK-293 cells by transient transfection. 24 h after transfection cells were serum starved for 18 h and then stimulated with either IGF (100 ng/ml 5 min), sorbitol (0.5 M for 30 min), AICar (2 mM for 1 hour), UV-C (200Jm2 followed by 1 hour incubation at 37°C), hydrogen peroxide (1 mM for 1 hour), PMA (400 ng/ml for 15 min), anisomycin (10 μg/ml for 1 hour) or left unstimulated. Cells were lysed and Pim kinase activity measured by immunoprecipitation assays as described in the methods. B) GST-Bad was transfected into HEK-293 cells along with either empty pCMV5 vector or FLAG-Pim-1, 2, or 3 expression constructs. 24 h after transfection cells were serum starved for 18 h and then left unstimulated or stimulated with either PMA (400 ng/ml for 15 min) or forskolin (20 μM for 30 min). Cells were then lysed and extracts immunoblotted for Bad phosphorylated on Ser112, Ser136 or Ser155, total Bad and FLAG (to monitor Pim-1, 2 or 3 expression). C) GST-Bad was transfected into HEK-293 cells with empty pCMV5 vector or active or kinase dead FLAG-Pim-1-3 expression constructs. 24 h after transfection cells were serum starved for 18 h and were then lysed and extracts immunoblotted for Bad phosphorylated on Ser112, Ser136 or Ser155, total Bad and FLAG (to monitor Pim-1, 2 or 3 expression).

To examine the regulation of Pim-1, 2 and 3 in cells, FLAG tagged Pim kinases were expressed in HEK-293 cells by transient transfection. Endogenous levels of Pim-1, 2 or 3 could not be detected in HEK-293 cells with the antibodies available. After serum starvation the cells well treated with various stimuli and the activity of the Pim kinases determined after immunoprecipitation with an anti-FLAG antibody. All three of the Pim kinases were found to be active in serum starved cells, and their activity was not further increased by stimulation of the cells with mitogenic signals (PMA or IGF), cellular stress (anisomycin, sorbitol, UV-C or hydrogen peroxide) or AICar, an activator of AMPK (Fig 3A).

GST-Bad expressed by transient transfection of serum starved HEK-293 cells, in the absence of the co-expression of any kinase, was found to be phosphorylated on Ser136, bur not Ser112 or Ser155, as judged by immunoblotting using antibodies specific for the various phosphorylation sites. Consistent with previous studies [33], stimulation of the cells with PMA resulted in a modest increase in Ser112 phosphorylation whilst forskolin resulted in increases in Ser155 phosphorylation and, to a lesser extent, Ser136 (Fig 3B). Co-expression of Pim-1 and Bad resulted in increased phosphorylation of Ser136 and phosphorylation of Ser112 and Ser155, when compared to Bad expressed in the absence of Pim-1. Similar results were also obtained for co-expression of Pim-2 or Pim-3, although Pim-2 had a reduced effect on Ser136 phosphorylation in Bad compared to co-expression with Pim-1 and Pim-3 (Fig 3B). The phosphorylation of Bad by Pim kinases was not further increased by PMA stimulation, although Ser155 phosphorylation was further increased by Forskolin stimulation. The phosphorylation of Bad was dependent on the kinase activity of Pim kinases, as co-expression of kinase dead versions of Pim-1, 2 or 3 and Bad did not result in the phosphorylation of Bad (Fig 3C).

**Figure 4:**
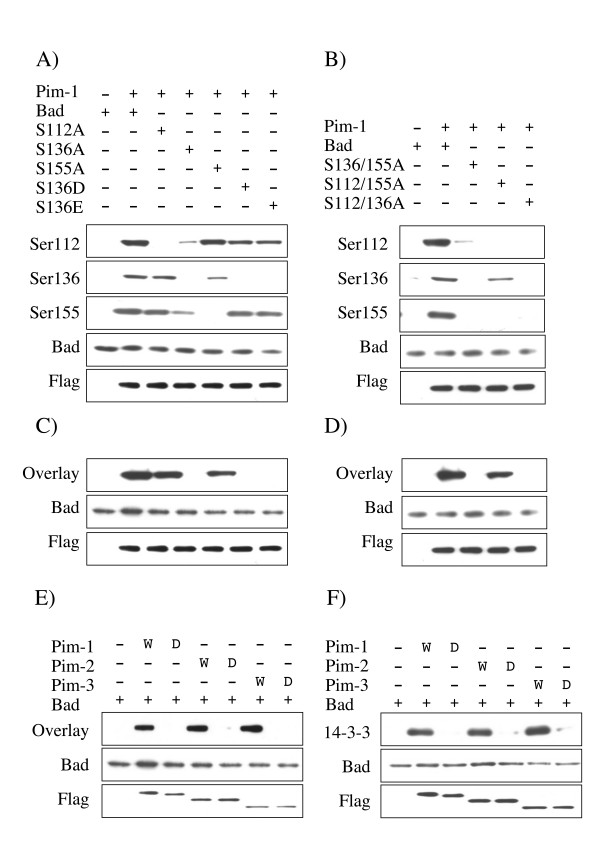
**Ser136 is a critical site to allow phosphorylation of Bad by Pim in cells**. A) GST-Bad, or Ser112Ala, Ser136Ala, Ser155Ala, Ser136Asp or Ser136Glu mutants of GST-Bad were transfected into HEK-293 cells along with either empty pCMV5 vector or a FLAG-Pim-1 expression constructs. 24 h after transfection cells were serum starved for 18 h and were then lysed and extracts immunoblotted with antibodies against Bad phosphorylated on Ser112, Ser136 or Ser155, total Bad or FLAG. B) GST-Bad, or Ser136/155Ala, Ser112/155Ala or Ser112/136Ala mutants of GST-Bad were transfected into HEK-293 cells with either empty pCMV5 vector or FLAG-Pim-1 expression constructs. 24 h after transfection cells were serum starved for 18 h and lysed and extracts immunoblotted with antibodies against Bad phosphorylated on Ser112, Ser136 or Ser155, total Bad or FLAG. C) GST-Bad, or Ser112Ala, Ser136Ala, Ser155Ala, Ser136Asp or Ser136Glu mutants of GST-Bad were transfected into HEK-293 cells along with either empty pCMV5 vector or a FLAG-Pim-1 expression constructs. 24 h after transfection cells were serum starved for 18 h and were then lysed and extracts immunoblotted for total Bad or FLAG (to monitor expression). Extracts were also run on SDS polyacrylamide gels, transferred to nitrocellulose and 14-3-3 overlays performed as described in the methods. D) GST-Bad, or Ser136/155Ala, Ser112/155Ala or Ser112/136Ala mutants of GST-Bad were transfected into HEK-293 cells with either empty pCMV5 vector or FLAG-Pim-1 expression constructs. 24 h after transfection cells were serum starved for 18 h and lysed and extracts immunoblotted for total Bad or FLAG (to monitor expression). Extracts were also run on SDS polyacrylamide gels, transferred to nitrocellulose and 14-3-3 overlays performed as described in the methods. E) GST-Bad was transfected into HEK-293 cells along with either empty pCMV5 vector or active (W) or kinase dead (D) FLAG-Pim-1, 2, or 3 expression constructs. 24 h after transfection cells were serum starved for 18 h and were then lysed and extracts immunoblotted for total Bad or FLAG (to monitor Pim-1, 2 or 3 expression). Extracts were also run on SDS polyacrylamide gels, transferred to nitrocellulose and 14-3-3 overlays performed as described in the methods. F) As E except that GST pull downs were performed on the extracts and immunoblotted for 14-3-3 as described in the methods.

The ability of Pim-1 to phosphorylate Bad in which one of the phosphorylation sites had been mutated was also determined by co-expression. As expected the mutation of Ser112 to alanine prevented phosphorylation of this site by Pim-1, but did not significantly affect the phosphorylation of either Ser136 or Ser155 (Fig 4A). Similarly, mutation of Ser155 prevented phosphorylation of this site by Pim-1 but did not significantly affect phosphorylation of Ser112. Surprisingly mutation of Ser155 to alanine did cause a reduction in the level of Ser136 phosphorylation by Pim-1 (Fig 4A) and a similar reduction in Ser136 phosphorylation was also seen in Ser112/Ser155Ala mutants (Fig 4B). Mutation of Ser136 to alanine not only blocked phosphorylation of Ser136 by Pim-1, but also greatly reduced Ser112 and Ser155 phosphorylation (Fig 4A). Mutation of Ser136 to an acidic residue to mimic phosphorylation was able to restore phosphorylation of Ser112 and Ser155 (Fig 4A).

Similar effects of the mutants were also seen in the absence of co-expressed Pim-1, when cells were stimulated with agents to induce the phosphorylation of specific sites within Bad. PMA promotes Bad Ser112 phosphorylation, probably via RSK, however this was prevented by Ser136 mutation. Forskolin stimulated Ser155 phosphorylation via PKA, and this was also prevented by mutation of Ser136 [see Additional file 1].

### Pim promotes 14-3-3 binding to Bad via phosphorylation of Ser136 and dissociation from Bcl-X_L_

Phosphorylation of a protein can create binding sites for 14-3-3 proteins, and Ser112 and 136 in Bad lie in a potential consensus 14-3-3 motif [38]. Ser155 does not lie in a consensus 14-3-3 binding motif, however *in vitro *phosphorylation of Bad by PKA (which predominantly phosphorylates Ser155) does promote 14-3-3 interaction [33]. To test the ability of Bad to interact with 14-3-3, GST-Bad was co-expressed in HEK-293 cells with Pim-1, and 14-3-3 overlay assays were used to determine if Bad could bind 14-3-3 proteins. In the overlay assay, recombinant 14-3-3 protein was used to detect the presence of proteins on a Western blot capable of binding 14-3-3. GST-Bad isolated by glutathione-Sepharose pull downs from the cell lysates was tested for its ability to bind to 14-3-3. In the absence of Pim-1 co-expression, GST-Bad was not significantly phosphorylated on Ser112, 136 or 155 and did not bind 14-3-3 in the overlay assay (Fig 4A and 4C). Co-expression of Pim-1 however resulted in phosphorylation of all three sites and promoted interaction of the GST-Bad with 14-3-3 in the overlay assay. Mutation of either Ser112 or Ser155 to alanine resulted in a modest reduction in the ability of the Pim-1 phosphorylated GST-Bad to interact with 14-3-3 proteins in the overlay assay (Fig 4C). As expected, mutation of Ser136 to alanine, which also blocks the phosphorylation of Ser112 and Ser155, completely blocked the ability of GST-Bad to interact with 14-3-3 proteins. Mutation of Ser136 to an acidic residue also blocked the ability of Bad to bind to 14-3-3 proteins in the overlay assay, even though Pim-1 was able to phosphorylate both Ser112 and Ser155 in the Ser136Asp or Ser136Glu Bad mutants (Fig 4C). These data suggest that Ser136 is the major 14-3-3 binding site. Consistent with this, a double mutant of both Ser112 and Ser155 to alanine, which was phosphorylated on Ser136 (Fig 4B), was still able to interact with 14-3-3 proteins in the overlay assay (Fig 4D). Similar to Pim-1, when Pim-2 or Pim-3 were co-expressed with GST-Bad, they were also able to promote 14-3-3 binding in the overlay assay (Fig 4E). To test if Pim kinases promoted the interaction of Bad and 14-3-3 proteins in cells, Pim kinases and GST-Bad were co-expressed in HEK-293 cells and the GST-Bad isolated by glutathione-Sepharose pull downs and blotted for the presence of endogenous 14-3-3 proteins using a pan 14-3-3 isoform antibody (Fig 4F). In the absence of Pim kinase co-expression, 14-3-3 proteins were not detected in the GST-Bad pull downs, however expression of Pim-1, 2 or 3 was found to promote the binding of 14-3-3 to GST-Bad in cells (Fig 4F). The ability of Pim kinases to promote the interaction of Bad and 14-3-3 was dependent on their kinase activity, as co-expression of kinase dead Pim-1, 2 or 3 did not promote 14-3-3 binding as judged by either co-precipitation or 14-3-3 overlay assays (Fig 4E and 4F).

Phosphorylation of Bad has been shown to prevent an interaction with Bcl-X_L _[39]. The ability of Pim kinases to prevent the interaction of GST-Bad with endogenous Bcl-X_L _was tested in pull down experiments. HeLa cells were used for these experiments as these cells express detectable levels of endogenous Bcl-X_L_.

**Figure 5:**
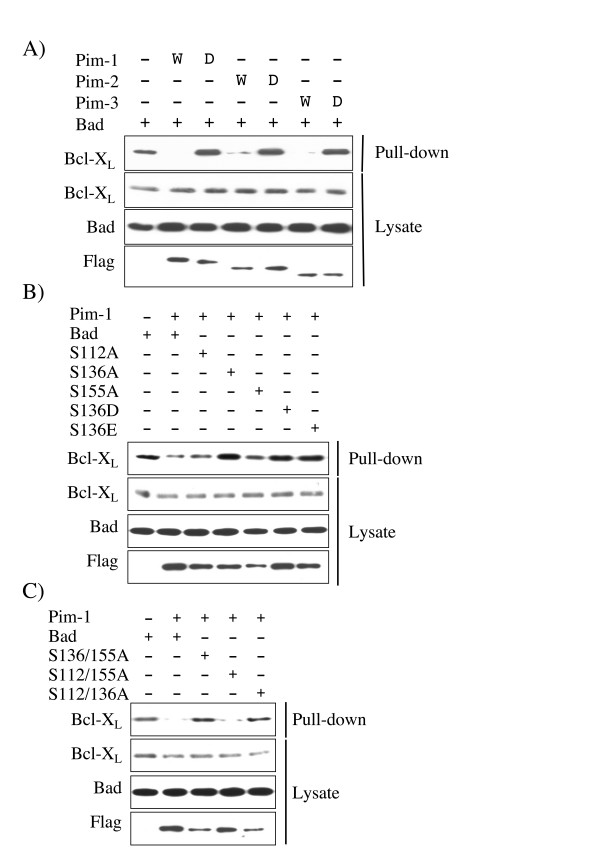
**Phosphorylation of Bad by Pim prevents Bcl-X_L _binding**. A) GST-Bad was transfected into HeLa cells along with either empty pCMV5 vector or active (W) or kinase dead (D) FLAG-Pim-1, 2, or 3 expression constructs. 24 h after transfection cells were serum starved for 18 h and were then lysed. GST pull downs were then performed on the extracts and imuunoblotted for Bcl-X_L _associating with the GST-Bad. Extracts were also immunoblotted with antibodies against total Bcl-X_L_, total Bad or FLAG. B) GST-Bad, or Ser112Ala, Ser136Ala, Ser155Ala, Ser136Asp or Ser136Glu mutants of GST-Bad were transfected into HEK-293 cells along with either empty pCMV5 vector or a FLAG-Pim-1 expression constructs. 24 h after transfection cell were serum starved for 18 h and were then lysed. GST pull downs were then performed on the extracts and immunoblotted for Bcl-X_L _associating with the GST-Bad. Extracts were also immunoblotted with antibodies against total Bcl-X_L,_, total Bad and FLAG. C) GST-Bad, or Ser136/155Ala, Ser112/155Ala or Ser112/136Ala mutants of GST-Bad were transfected into HEK-293 cells with either empty pCMV5 vector or FLAG-Pim-1 expression constructs. 24 h after transfection cells were serum starved for 18 h and lysed. GST pull downs were then performed on the extracts and immunoblotted for Bcl-X_L _associating with the GST-Bad. Extracts were also immunoblotted with antibodies against total Bcl-X_L_, total Bad or FLAG.

As expected, GST-Bad, which was not significantly phosphorylated in the absence of kinase co-expression, was able to interact with Bcl-X_L _in HeLa cells. Co-expression of Pim-1, 2 or 3 resulted in phosphorylation of GST-Bad and reduced the interaction of GST-Bad with Bcl-X_L_. This was dependent on the kinase activity of Pim, as co-expression of kinase dead Pim mutants did not have this effect (Fig 5A). Similar to the 14-3-3 interaction, Ser136 appeared to be the critical residue involved in this process. Mutation of Ser112, Ser155 (Fig 5B), or both Ser112 and Ser155 to alanine (Fig 5C) in Bad did not affect the ability of Pim-1 to prevent the interaction of GST-Bad and Bcl-X_L_. Mutation of Ser136 in Bad to alanine (which blocked phosphorylation of all three sites) or an acidic residue (which did not affect Ser112 or Ser155 phosphorylation) blocked the ability of Pim-1 to promote the dissociation of GST-Bad and Bcl-X_L _(Fig 5B).

## Discussion

Pim-1, 2 or 3 were found to be able to phosphorylate Bad *in vitro *and in cells. Pim-2 was found to be the most specific of the Pim kinases for Ser112 of Bad *in vitro*, although it was also able to phosphorylate Ser155 both *in vitro *and when over-expressed in HEK-293 cells. Pim-2 was a poor Ser136 kinase *in vitro *and, consistent with this, was the least effective of all the Pim kinases at phosphorylating this site in cells. In agreement with this Pim-2 has previously been described to phosphorylate Ser112 [20,21]. Interestingly, the phosphorylation of Ser136 and Ser155 by over-expression of Pim-2 has been suggested to be dependent on the cell line used [21]. Both previous results and our results suggest this may reflect different over-expression levels of Pim-2 achieved in the different cell types used. Pim-1 was able to phosphorylate Ser112, Ser136 and Ser155 in Bad, however phosphorylation of Ser112 was significantly more efficient than phosphorylation of the other sites *in vitro*. Pim-1 has previously been found to phosphorylate Ser112 in Bad, although its ability to phosphorylate Bad on other sites has not previously been reported [22]. The ability of Pim-3 to phosphorylate Bad has not previously been addressed. Here we report that Pim-3 is also able to phosphorylate Ser112 in Bad. Interestingly Pim-3 was the most effective kinase *in vitro *for Ser136 of Bad. Unlike Pim-1 or Pim-2, which demonstrated a marked preference for Ser112, Pim-3 was also effective at phosphorylating Ser136, and to a lesser extent Ser155, when Bad protein was used as an *in vitro *substrate (Table 1). In addition Pim-3 was also able to phosphorylate other sites in Bad including Ser170 *in vitro*. This site has been reported to be phosphorylated *in vivo*, although the kinase responsible was not identified in the initial study [36]. Mutagenesis of Ser170 in Bad has suggested that phosphorylation of this site, like phosphorylation of Ser112, 136 and 155, inhibits the pro-apoptotic activity of Bad [36]. Phosphorylation of this site by Pim-3 would therefore be consistent with the pro-survival function of Pim-3. To the best of our knowledge, this study is the first to report a Pim-3 cellular substrate.

The interaction between the phosphorylation sites in Bad and their roles in Bad function is complex. Phosphorylation of Ser136 appears to be required for the phosphorylation of Ser112 and Ser155 to occur when Bad is over-expressed in cells. Similarly, Ser136 appears to be the major 14-3-3 binding site in Bad (Fig 4) [38,40], while Ser155 is suggested to be required for binding to Bcl-X_L _[41]. Despite this, phosphorylation of endogenous Bad on Ser136 has proven hard to demonstrate *in vivo*. This may reflect the poor sensitivity of the antibodies available, however it has also been suggested that this site may not be physiologically important [42]. In contrast to this, studies using transfected Bad have suggested that phosphorylation of this site is essential for the phosphorylation of Ser112 and Ser155 and 14-3-3 binding, which are required to inhibit the apoptotic activity of Bad [38,40]. Studies on Ser112 have suggested that this site may be a 'gatekeeper' site, and that dephosphorylation of this site is required to recruit a phosphatase that then dephosphorylates Ser136 and Ser155 [43]. An extension of this idea lead to the proposal that Pim-1 only directly phosphorylates Ser112, and that an increase in Ser136 phosphorylation when Pim-1 was over-expressed was due to a decrease in the rate of Ser136 dephosphorylation [22]. Whilst this cannot be completely excluded, it is not consistent with our data that Pim-1 can promote the phosphorylation of a Ser112Ala Bad mutant in cells. The interplay between the different Bad phosphorylation sites is thus very complex and further work is required to completely understand these mechanisms. An added degree of complexity is the number of physiological kinases for Bad, including Pim-1-3, PKB, PAK, RSK, PKA [26-35] and possibly other kinases potentially playing a role. The exact context in which Pim phosphorylates Bad *in vivo *may therefore depend on both cell type and stimulus and remains to be fully understood.

The ability of Pim kinases to phosphorylate Bad is consistent with the suggested role of these kinases in pro survival or proliferation pathways. The finding that all three Pim kinases can phosphorylate similar sites in Bad could in part explain the redundancy between Pim kinases in providing a cellular survival signal. The knockout of Pim kinase might therefore be expected to have a severe phenotype, however a triple knockout of Pim-1, 2 and 3 has recently been reported. Surprisingly these mice were found to be viable, and while they were smaller in size than wild type mice they displayed no obvious health problems [44]. These mice did however show reduced proliferation of some haematopoietic cells in response to growth factors. Interestingly, a triple knockin mutation of Bad (resulting in the mutation of Ser112, 136 and 155 to alanine) in mice was also found to be viable and did not show increased levels of apoptosis in tissues under normal conditions [45]. These mice also displayed reduced numbers of T and B cells and reduced cytokine induced survival of these cells *in vitro*, a phenotype with some similarity to that of the triple Pim kinase knockout.

## Conclusion

We present here biochemical evidence that all three isoforms of Pim kinase are able to phosphorylate Bad. The major site of Bad phosphorylation for all three isoforms of Pim kinase is Ser112. However, we also demonstrate that Pim kinases are able to phosphorylate Bad to a lesser extent on Ser136 and Ser155 *in vitro*. Interestingly Pim-3 exhibits less specificity for Ser112 than Pim-1 or Pim-2, and was the most effective pim isoform for Ser136 and Ser155 phosphorylation in Bad *in vitro*. We also provide evidence that Pim kinases may modulate the phosphorylation of Bad on Ser112, Ser136 and Ser155 in HEK-293 cells.

## Methods

### Plasmids

The coding region for an amino-terminal extended version of human Pim-1 (NCBI Acc. NM_002648) predicted to be synthesised by alternative translation initiation at an upstream CUG codon [46] was amplified from IMAGE consortium EST clone 4591723 using primer 5'-gcgaattcgccaccatggactacaaggacgacgatgacaagctgccgcacgagccccacgag-3' incorporating a 5' FLAG tag sequence and primer 5'- gcgaattcctatttgctgggccccggcgac-3'. The PCR product was ligated into pCR2.1-TOPO vector (Invitrogen), sequenced, and sub-cloned as an *Eco*R1-*Eco*R1 insert into the pCMV5 expression vector [47]. The same coding region was cloned into expression vector pEBG2T [48] without the 5' FLAG tag sequence using appropriate primers as a *Spe*1-*Spe*1 insert. The coding region for an amino-terminal extended version of human Pim-2 (NCBI Acc. NM_006875.2) predicted to be synthesised by alternative translation initiation at an upstream CUG codon was amplified from IMAGE consortium EST clone 3913552 using primer 5'- gcgaattcgccaccatggactacaaggacgacgatgacaagctggcgcgcgcggcgaatctcaac-3' incorporating a 5' FLAG tag sequence and primer 5'- gcggatccttagggtagcaaggaccaggccaaag-3'. The PCR product was ligated into pCR2.1-TOPO vector, sequenced, and sub-cloned as an *Eco*R1-*Bam*H1 insert into the pCMV5 expression vector. The same coding region was cloned into expression vector pEBG2T without the 5' FLAG tag sequence using appropriate primers as a *Bam*H1-*Not*1 insert. Nucleotides 125–1455 (stop codon of CDS) of murine Pim-3 (NCBI Acc. NM_145478) mRNA were amplified from IMAGE consortium EST clone 4924687 using primer 5'-gcgaattcgagcgtccggcttccccag-3' and primer 5'-gcgaattctcacttgtcatcgtcgtccttgtagtccaagctctcactgctggaagtg-3' incorporating a FLAG tag sequence at the 3' end. The PCR product was ligated into pCR2.1-TOPO vector, sequenced, and sub-cloned as an *Eco*R1-*Eco*R1 insert into expression vector pCMV5. The same region of Pim-3 mRNA was cloned (lacking the Pim-3 stop codon) with a C-terminal GST sequence (generated by overlap PCR) into pCMV5 as a Kpn1/HindIII insert. The Pim-3 sequence was also cloned with an N-terminal His Tag in to a pFastBAC vector for expression in Baculovirus expression. Kinase dead versions of the Pim-1, Pim-2 and Pim-3 expression constructs were generated by mutating the 'DFG' motif to 'AFG' in each case using the QuikChange Site-Directed Mutagenesis Kit (Stratagene). A cDNA encoding full-length murine Bad was amplified from the pGEX Bad construct described in [33] using primer 5'- gcggatccatgggaaccccaaagcagccctc-3' and primer 5'- gtgcggccgctcactgggagggggtggagcc-3'. The PCR product was ligated into pCR2.1-TOPO vector, sequenced, and sub-cloned as a *BamH*1-*Not*1 insert into expression vector pEBG6P-1 (pEBG2T containing a PreScission Protease site). S112A, S155A, S136A, S136D and S136E mutants were generated using the QuikChange Site-Directed Mutagenesis Kit (Stratagene).

### Cell culture

HEK-293 cells were maintained in DMEM containing 10% FBS (Sigma), 2 mM L-glutamine, 50 units/ml penicillin G and 50 μg/ml streptomycin (Invitrogen). Cells were transfected using PEI as described [49]. Prior to stimulation and analysis cells were starved in DMEM lacking serum for eighteen hours at 37°C and 5% CO_2_. Cells were stimulated with IGF (100 ng/ml 5 min), sorbitol (0.5 M for 30 min), AICar (2 mM for 1 hour), UV-C (200 Jm^2^/10 sec followed by 1 hour incubation at 37°C), hydrogen peroxide (1 mM for 1 hour), PMA (400 ng/ml for 15 min), anisomycin (10 μg/ml for 1 hour) or Forskolin (20 μM for 30 min). Cells were lysed in 50 mM Tris-HCl pH 7.5, 1 mM EGTA, 1 mM EDTA, 1 mM sodium orthovanadate, 50 mM sodium fluoride, 1 mM sodium pyrophosphate, 0.27 M sucrose, 1% (v/v) Triton X-100, 0.1% (v/v) 2-mercaptoethanol. Lysates were clarified at 13,000 rpm for 5 min at 4°C and the supernatants quick frozen in liquid nitrogen and stored at -80°C until used.

### Peptide kinase assay

For *in vitro *kinase assays 1 unit of activity was defined as the amount of kinase that catalysed the phosphorylation of 1 nmol of substrate in 1 min into the peptide RSRMSSYPDRG at a 30 μM peptide concentration. To determine the ability of Pim kinases to phosphorylate peptides derived from Bad, each peptide substrate was used at the concentrations indicated and incubated with 10 mU of kinase in a 50 μl volume for 10 min at 30°C in 10 mM MgCl_2 _– 0.1 mM [γ^32^P]-ATP, 50 mM Tris-HCl, pH 7.5, 10 mM EGTA, 0.1% 2-mercaptoethanol and 2.5 μM PKI. Reactions were terminated by spotting on to p81 paper, followed by immersion in 50 mM ortho-phosphoric acid. All papers were then washed three times in 50 mM ortho-phosphoric acid to remove unincorporated ATP, once in acetone and then dried and the ^32^P incorporation was determined by Cerenkov counting. The peptides used were RSRMSSYPDRG (corresponding to Ser112), RGSRSAPPNL (corresponding to Ser136) and RELRRMSDEFEGS (corresponding to Ser155).

To determine the activity of Pim kinases expressed in Hek-293 cells, FLAG-Pim kinase was immunoprecipitated from 50 μg of soluble cell lysate using an anti FLAG antibody (Sigma) and assayed by standard protocols [50]. Briefly, immunoprecipitates were washed twice in 0.5 M NaCl, 50 mM Tris-HCl pH 7.5, 0.1 mM EGTA and 0.1% (v/v) 2-mercaptoethanol and once in 50 mM Tris-HCl pH 7.5, 0.1 mM EGTA and 0.1% (v/v) 2-mercaptoethanol. Precipitates were then resuspended in 35 μl of reaction buffer (containing Tris/HCl pH7.5, EGTA, PKI and RSRMSSYPDRG substrate peptide,), and the reaction was started by the addition of 10 μl 50 mM MgAc, 0.5 mM [γ^32^P]-ATP and incubated at 30°C for 15 min. Final concentrations of reagents in the assay were 50 mM Tris/HCl pH7.5, 0.1 mM EGTA 0.1% 2-mercaptoethanol 2.5 μM PKI (an inhibitor of PKA), 30 μM substrate peptide peptide, 10 mM MgAc and 0.1 mM [γ^32^P]-ATP. Reactions were stopped by transfer onto P81 paper and washing in 75 mM orthophosphoric acid.

### *In vitro *Bad phosphorylation

Human Pim-1 and Pim-2 were cloned with an N-terminal GST-tag and expressed in HEK-293 cells. Murine Pim-3 was cloned with an N-terminal His-tag and expressed in a Baculovirus expression system using *Sf*9 insect cells. Murine Bad was cloned with an N-terminal GST-tag and expressed in bacteria. All recombinant proteins were purified using standard affinity chromatography techniques on glutathione or Ni-NTA sepharose as appropiate. Purified recombinant Pim-kinases (10 mU) were incubated with 2 μg of recombinant Bad over the indicated time period in 50 mM Tris-HCl, pH 7.5, 10 mM EGTA, 0.1% 2-mercaptoethanol, 2.5 μM PKI, 10 mM MgCl_2 _and 0.1 mM [γ^32^P]-ATP. Reactions were stopped by the addition of SDS loading buffer and samples analysed on a 4–12% Novex gel (Invitrogen). Protein was detected by Colloidal blue staining and the appropriate band was excised and the amount of ^32^P incorporation was determined by Cerencov counting.

### Phosphorylation site identification

Samples were run on a 4–12% polyacrylamide gel, and the substrate band was excised and the amount of ^32^P incorporated determined by Cerenkov counting. For phospho site mapping, the protein was reduced with dithiothreitol, alkylated with iodoacetamide and digested with trypsin. The resulting peptides were applied to a Vydac 218TP5215 C18 column equilibrated in 0.1% trifluoroacetic acid and the column developed with a linear gradient of acetonitrile/0.1% trifluoroacetic acid at a flow rate of 0.2 ml/min while collecting 0.1 ml fractions. ^32^P radioactivity was recorded with an on-line monitor. Phosphorylation site mapping was performed essentially as described previously [51]. Identification of ^32^P labelled peptides was performed by MALDI-TOF and MALDI-TOF-TOF mass spectrometry on an Applied Biosystems 4700 using a matrix of 10 mg/ml alpha-cyanocinnamic acid in 50% acetonitrile/0.1% trifluoroacetic acid/10 mM ammonium phosphate. Sites of phosphorylation within the peptides were determined by a combination of MALDI-TOF-TOF mass spectrometry and solid phase Edman sequencing. Solid phase sequencing was performed on an Applied Biosystems Procise 494C after coupling the peptide covalently to a Sequelon-arylamine membrane and measuring by Cerenkov counting the ^32^P radioactivity released after each cycle.

### GST-Bad pull down

Lysates were incubated with glutathione-sepharose for 2 hours at 4°C. Immunoprecipitates were pelleted by centrifugation for 1 min at 13,000 g and washed twice in 50 mM Tris-HCl, pH 7.5, 500 mM NaCl, 0.1 mM EGTA and 0.1 % 2-mercaptoethanol and once in 50 mM Tris-HCl, pH 7.5, 0.1 mM EGTA and 0.1 % 2-mercaptoethanol. Protein complexes were eluted in SDS sample buffer.

### Immunoblotting

Soluble cell extract was run on 4–12% Novex gels (Invitrogen) and transferred onto nitrocellulose membranes and blotted using standard techniques. Antibodies that recognise p-Bad (Ser112), p-Bad (Ser 136), pBad (Ser155) and Bcl-X_L _were from Cell Signalling Technology. An antibody that detected total Bad protein was raised in-house. The Flag antibody was from Sigma and HRP conjugated secondary antibodies were from Pierce (Cheshire, UK) and blots were developed using the enhanced ECL reagent (Amersham, Buckinghamshire, UK).

### 14-3-3 overlay assays

14-3-3 overlay assays were performed as described [52]. Briefly, proteins from cell extracts or GST pull downs were separated on 4–12% Novex gels (Invitrogen) and transferred to nitrocellulose membrane. Membranes were then treated as for immunoblots. Digoxygenin (DIG)-labelled 14-3-3 proteins were incubated with the membranes in place of the primary antibody, followed by incubation with a secondary anti-DIG horseradish peroxidase antibody. Blots were developed using the enhanced ECL reagent (Amersham, Buckinghamshire, UK).

## Abbreviations

PIM (proviral integration of MMLV), PKA (cAMP-dependent protein kinase), RSK (p90 ribosomal S6 kinase), PAK (p21 activated protein kinase), PKI (cAMP-dependent protein kinase inhibitor peptide), GST (glutathione S-transferase), ECL (enhanced chemiluminescence), IGF (insulin-like growth factor), PMA (phorbol 12-myristate 13-acetate), HEK (human embryonic kidney), DMEM (Dulbecco's modified eagles medium).

## Authors' contributions

AM was involved in all aspects of this study and was responsible for most of the experimental work. Mass spectrometry analysis was provided by DC and molecular biology assistance was provided by RT. HM and JH supervised the provision of protein and antibody reagents. JSCA and AM were responsible for co-ordinating the study and drafting the paper.
